# Effect of dexamethasone on quality of life in children with acute lymphoblastic leukaemia: a prospective observational study

**DOI:** 10.1186/1477-7525-6-103

**Published:** 2008-11-26

**Authors:** Machteld AG de Vries, Raphaële RL van Litsenburg, Jaap Huisman, Martha A Grootenhuis, A Birgitta Versluys, Gert Jan L Kaspers, Reinoud JBJ Gemke

**Affiliations:** 1 Department of paediatrics and division of oncology-haematology, VU University Medical Centre, Amsterdam, The Netherlands; 2 Department of medical psychology, VU University Medical Centre, Amsterdam, The Netherlands; 3 Paediatric Psychosocial Department, Emma Children's Hospital, Amsterdam, The Netherlands; 4 Department of paediatrics, division of oncology-haematology, Wilhelmina Children's Hospital University Medical Centre, Utrecht, The Netherlands

## Abstract

**Background:**

Glucocorticoids are important in the treatment of childhood acute lymphoblastic leukaemia (ALL). However, cyclic administration of high dose glucocorticoids may cause rapid and substantial changes in quality of life (QoL). The maintenance phase of the Dutch ALL-9 protocol consisted of alternating two weeks on and five weeks off dexamethasone (6 mg/m^2^/day). The present study was performed to assess the effect of dexamethasone on QoL during treatment for ALL according to this protocol.

**Methods:**

In a multicentre prospective cohort study, QoL was assessed halfway (T1) and at the end of the two-year treatment (T2). A generic (Child Health Questionnaire) and disease specific (PedsQL™ cancer version) QoL questionnaire were used to assess QoL in two periods: on and off dexamethasone, respectively.

**Results:**

41 children (56% males) were evaluated, mean age at diagnosis was 5.6 years. The CHQ physical and psychosocial summary scores were significantly lower than population norms. At T1 and T2, overall QoL showed no significant change. However, regarding specific domains (pain, cognitive functioning, emotion/behaviour and physical functioning) QoL decreased over time. QoL was significantly more impaired during periods on dexamethasone.

**Conclusion:**

Dexamethasone was associated with decreased QoL. At the end of treatment, reported QoL during dexamethasone deteriorated even more on certain scales (pain, cognitive functioning, emotion/behaviour and physical functioning). Knowledge of the specific aspects of QoL is essential to improve counselling and coping in paediatric oncology. Adverse effects of specific drugs on QoL should be taken into account when designing treatment protocols.

## Background

Acute Lymphoblastic Leukaemia (ALL) is the most common form of childhood cancer. Over the past decades survival following treatment for childhood ALL has improved substantially, now reaching about 80%. With increased survival rates, issues concerning the Quality of Life (QoL) of these children become increasingly relevant. This is reflected in the steady rise in studies concerning QoL. Most of these studies have focused on the follow-up of childhood cancer survivors and, to a lesser extend, on children during active treatment. It seems most children experience reduced QoL during and after treatment. [1-4] More attention to treatment related factors of decreased QoL may provide health-care workers with specific tools to address these issues.

Glucocorticoids are an important drug in current ALL therapy. They induce apoptosis and glucocorticoid responsiveness is an early indicator of response to chemotherapy in general. Most treatment protocols include glucocorticoids during induction, reinduction and/or maintenance therapy. Dexamethasone has proven superior over prednisone, reducing relapse rate and improving event free survival [5-8], although there is evidence it might cause more side-effects. [7]

Studies on glucocorticoid-related psychosocial morbidity, although often clinically evident, in children are sparse. A review on this subject by Stuart et al. [9] in 2005, yielded ten larger studies, only two of which concerned childhood ALL, and several case-reports. Possible steroid-related side effects include emotional lability, anxiety, aggressive behaviour, hyperactivity, depression [10,11], problems with concentration, excessive eating [12], increase in pain [13] and sleep disorders [14]. These side-effects can seriously affect QoL. Glucocorticoids are likely to contribute to the rapid and intensive changes in QoL, mood and behaviour during ALL therapy. [13,15]

The aim of this study was to assess the effect of dexamethasone on QoL during maintenance treatment in children with ALL. Based on clinical experience and previous studies, it was hypothesized that QoL was more impaired during periods on dexamethasone than during periods without dexamethasone. [13,15]

## Methods

### Patients

A prospective, multicentre cohort study was designed. Children from three different Dutch centres were enrolled (WKZ Utrecht, AMC Amsterdam and VU University Medical Centre Amsterdam). All children between the ages of 2 and 18 years on active treatment according to the Dutch Childhood Oncology Group (DCOG) ALL-9 protocol were eligible. Parents who were not fluent in Dutch were excluded. Children with an important pre-existing condition (e.g. Down syndrome) were excluded because of a potentially different baseline quality of life.

This treatment protocol was open for inclusion from 1996 to 2004. Children with one of the following characteristics at diagnosis were stratified into the High Risk group (HR): initial leukocyte count >50 × 10^9^/l, presence of mediastinal enlargement, initial leukaemia of the central nervous system or testis, presence of t(9;22) or BCR-ABL, t(4;11) or 11q23 with MLL rearrangement and T-cell immunophenotype. All other children were classified as Non-High risk (NHR). Important differences in induction treatment between both risk groups consisted of total methotrexate dose (NHR 6000 mg/m^2 ^and HR 12000 mg/m^2^). The HR group received two additional intensification treatment blocks after induction. Maintenance treatment consisted of five weeks of mercaptopurine and methotrexate alternated with two weeks of 6 mg/m^2^/day of dexamethasone and weekly vincristine. Maintenance treatment was similar for both groups except for methotrexate, which was given intravenously for the HR group as opposed to orally for the NHR group, and frequency of intrathecal therapy (including age dependent methotrexate dose, NHR four times and HR seven times). Total duration of therapy was 109 weeks.

Inclusion into the study was halfway through treatment, whenever possible, allowing for two consecutive measurements: 12 months after the initial diagnosis (T1) and at the end of the two year treatment (T2). During 18 months (spring 2005 – fall 2006) all eligible patients were informed about the study at their paediatric oncology clinic. Questionnaires were sent by mail together with written information about the study and a stamped return envelope. Written informed consent was obtained prior to inclusion. If questionnaires were not returned within a month, patients were contacted by one of the researchers. At T1 and T2 QoL assessment tools were filled out for the most recent period on dexamethasone and the most recent period off dexamethasone. Since at the start of this study recruitment for ALL-9 had ceased and a new treatment protocol had already started, inclusion was maximized by including all children still treated according to ALL-9. For some children only measurement at T2 was possible, because they had already been treated for over a year when this study opened. The study was approved by the medical ethical review board.

### Measures

Parents (either father or mother) rated their children's QoL using both a generic and disease specific instrument. The Dutch version of the Child Health Questionnaire 50 items parent form (CHQ-PF50) is a generic QoL assessment tool and has shown good reliability and validity. [16,17] The CHQ has been used in several paediatric oncology studies. [3,4,18] This instrument covers the physical, emotional and social well-being of children and allows for two summary scores (physical and psychosocial). Items are scored using a four to six point Likert scale and converted to a 0 to 100 point continuum, with higher scores indicating better QoL. The original reference period of the CHQ (four weeks) was adjusted to suit the two week dexamethasone period. Dutch population norms are available and allow for a comparison with the Dutch healthy population. [16] The CHQ was designed for children five years and up. Although the Infant and Toddler Quality of Life Questionnaire would have been more appropriate for the younger children in our study sample [19], at the time of the design of our study, no validated Dutch version and norms were available.

The Paediatric Cancer Quality of Life Inventory 3.0™ Acute Cancer Version (PedsQL) is a cancer specific questionnaire that was translated into Dutch in close corroboration with the original author. The PedsQL cancer version has frequently been used in paediatric oncology studies. [20-22] It has proven a reliable and valid QoL assessment tool [23] with subscales for determining problems in relevant areas during cancer treatment such as pain, nausea, treatment and procedural anxiety, worry, cognitive problems, perceived physical appearance and communication. Items are scored using a four point Likert scale and reflect on the past week. Higher scores indicate better QoL. The scale incorporates age-specific questions and is also available in a parallel form for children from the age of five years onwards.

At T1 and T2 the questionnaires for assessment of both periods on and off dexamethasone, respectively, were sent in one mailing. Parents were instructed to assess both periods independently. Children aged 8 years or older were asked to do the same for the PedsQL only. Five to seven year olds were felt to be too young to participate, particularly since the questionnaires were sent to the family's homes and researchers were unable to coach the children. Although a child version of the CHQ is available and obtaining self-reports seems preferable whenever possible, only the PedsQL self-report was used to minimize patient burden.

### Statistics

The Statistical Package for Social Sciences (SPSS) for Windows version 12.0 was used for all the analyses. Differences in QoL subscale and total scores for periods on and off dexamethasone were compared using paired t-tests or Wilcoxon signed ranks tests. Change in QoL scores over time were also compared using paired t-tests or Wilcoxon signed ranks tests. The magnitude and meaning of the differences in QoL are represented as Cohen's effect size (*d*). Effect sizes are calculated as follows: [mean(a) – mean (b)/largest standard deviation score (SD)], this means that differences between groups are expressed in units of the largest within-group standard deviation. Effect size between 0.2 and 0.5 indicate a small effect, an effect size between 0.5 and 0.8 indicate a moderate effect, and effect sizes ≥ 0.8 represent a large effect. [24,25] Differences in QoL score between treatment groups were compared using a Mann-Whitney-U test. T-tests were used for comparison with CHQ Dutch population norms. Significance level was set at p < 0.05 for all analyses.

## Results

### Demographics

**Figure 1:**
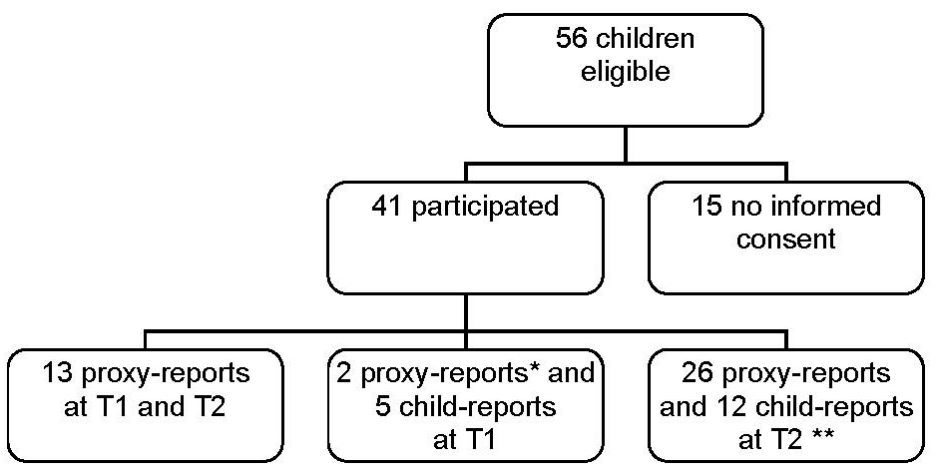
**Study participation**. * no participation at T2 because of: recurrence of leukaemia (n = 1) and not returning the questionnaires at T2 (n = 1). ** n = 2 were not treated with dexamethasone anymore because of serious corticosteroid related complications and only returned the off dexamethasone questionnaire.

A total of 56 children were eligible. All parents were invited to join this study, 41 (73%) eventually participated (see Figure 1). No information on the other 15 children was available, since no informed consent was obtained. Mean age at diagnosis was 5.6 years, 56% were male. 78% of all children were treated according to the Non-High risk protocol. There was no statistically significant difference in age or gender between the High and Non-High risk group (Table 1). At T1 only five child self reports were obtained, the other children were too young to fill out self-reports. These results were therefore omitted from statistical analysis. At the end of treatment, 12 self reports were returned. These results were taken into analysis.

**Table 1:** Demographics

	**Total**	**Non-High Risk**	**High Risk**	p
N	41	32 (78%)	9 (22%)	
Males (%)	23 (56%)	18 (78%)	5 (56%)	NS
Mean age (yrs) at diagnosis (SD)	5.6 (3.3)	5.8 (3.5)	4.9 (2.6)	NS

### PedsQL acute cancer version, parent-reports

Halfway as well as at the end of treatment, parents rated their child's overall QoL to be more impaired during periods on dexamethasone as compared to periods off dexamethasone. This also applied to most of the PedsQL subscales (see Table 2). The effect size of the difference in score between periods on and off dexamethasone on the pain subscale was 0.88 at T1 and 0.91 at T2, indicating more pain during the use of dexamethasone and representing a large effect. Moderate effect sizes were found for the total score (T1 *d *= 0.58; T2 *d *= 0.47) and the cognitive subscale (T1 *d *= 0.53; T2 *d *= 0.78). Comparing both time points (T1 and T2) overall QoL remained stable, except for the subscales of pain (p = 0.01; *d *= 0.44) and cognitive problems (p = 0.03; *d *= 0.42), for which the adverse effect of dexamethasone was more pronounced at T2. Problems in the area of perceived physical appearance worsened over time for the periods off dexamethasone (p = 0.03; *d *= 0.34).

**Table 2:** PedsQL™ 3.0 acute cancer module Parent Form

	T1 (n = 15)				T2 (n = 37)			
	Dexa + Mean (SD)	Dexa – Mean (SD)	Effect size^1^	p^2^	Dexa + Mean (SD)	Dexa – Mean (SD)	Effect size^1^	p^3^
Total	68.0 (15.6)	77.0 (8.5)	-0.58	0.01	66.2 (14.6)	73.1 (13.1)	-0.47	<0.01
Pain	53.3 (21.9)	72.6 (15.1)	-0.88	0.02	41.9* (26.2)	65.9 (21.8)	-0.91	<0.01
Nausea	74.5 (21.7)	75.0 (17.3)	0.02	NS	73.9 (22.4)	67.0 (22.1)	0.30	0.04
Procedural Anxiety	55.6 (33.7)	63.3 (27.4)	-0.23	0.03	71.2(29.3)	75.5 (24.2)	-0.15	NS
Treatment Anxiety	75.0 (30.7)	83.3 (25.0)	-0.27	0.04	80.6 (22.8)	84.5 (22.4)	-0.17	NS
Worry	86.1 (23.5)	87.2 (15.1)	-0.05	NS	69.4 (29.7)	76.4 (24.9)	-0.24	NS
Cognitive	66.2 (27.5)	80.8 (17.4)	-0.53	NS	54.6* (23.2)	72.8 (19.7)	-0.78	<0.01
Physical appearance	65.6 (28.3)	77.8 (19.3)	-0.43	0.03	62.1 (27.6)	69.3* (24.9)	-0.26	<0.01
Communication	62.0 (39.3)	75.1 (28.7)	-0.33	0.03	67.1 (28.5)	75.6 (24.2)	-0.30	<0.01

Higher scores indicate a better QoL

^1 ^Effect size = negative effect size indicates worse QoL in the group on dexamethasone. Positive effect size indicates better QoL in the group on dexamethasone: 0.2 ≤ *d *< 0.5 = small effect; 0.5 ≤ *d *< 0.8 = moderate effect; *d *≥ 0.8 = large effect.

^2 ^Dexa + versus Dexa - at T1, Wilcoxon signed ranks test

^3 ^Dexa + versus Dexa - at T2, T-test for paired samples

* significant difference T1 vs. T2

Except for children in the HR group, who experienced more cognitive problems (both on and off dexamethasone) at the end of treatment, there were no differences between both risk groups. Mean score at T2 on the cognitive subscale during dexamethasone was 34.0 (SD 24.0) for the HR group and 59.3 (SD 21.3) for the NHR group (p = 0.05). Off dexamethasone mean scores were 59.1 (SD 21.1) for the HR group and 77.8 (SD 17.1) for the NHR group (p = 0.03). Effect sizes were large (dexamethasone *d *= 1.05; off dexamethasone *d *= 0.89).

### PedsQL acute cancer version, child-reports

At T2, there was a non significant trend that children also judged their own overall QoL to be worse during periods on dexamethasone (see table 3). At T2 during dexamethasone, scores on the PedsQL were significantly lower for the subscales pain (p = 0.04; *d *= 0.70), worry (p = 0.02; *d *= 0.50) and cognition (p = 0.01; *d *= 0.67). Nausea was scored significantly better (p = 0.04), although the effect size was small (*d *= 0.15). Because of the small sample size, no statistically significant differences between parent and child rating of QoL could be demonstrated. For both periods on and off dexamethasone, there was a significant positive correlation between parent and child answers (on dexamethasone r = 0.76 [p < 0.001] and off dexamethasone r = 0.81 [p < 0.001]).

**Table 3:** PedsQL™ 3.0 acute cancer module Child Form at T2 (n = 12)

	Dexa + Mean (SD)	Dexa – Mean (SD)	Effect size^1^	p^2^
Total	67.7 (15.4)	73.6 (12.0)	-0.38	NS
Pain	45.8 (23.4)	67.0 (30.5)	-0.70	0.04
Nausea	75.0 (24.2)	71.4 (21.0)	0.15	0.04
Procedural Anxiety	77.8 (26.0)	78.0 (23.9)	-0.01	NS
Treatment Anxiety	86.8 (22.6)	88.7 (20.8)	-0.08	NS
Worry	62.5 (26.2)	75.6 (18.3)	-0.5	0.02
Cognitive	56.3 (24.9)	73.1 (15.5)	-0.67	0.01
Perceived physical appearance	60.4 (22.2)	63.7 (16.9)	-0.15	NS
Communication	72.2 (32.8)	71.3 (27.9)	-0.03	NS

Higher scores indicate a better QoL

^1 ^Effect size = negative effect size indicates worse QoL in the group on dexamethasone. Positive effect size indicates better QoL in the group on dexamethasone: 0.2 ≤ *d *< 0.5 = small effect; 0.5 ≤ *d *< 0.8 = moderate effect; *d *≥ 0.8 = large effect.

^2 ^Wilcoxon signed ranks test

### CHQ-PF50

The CHQ physical summary score (PhS) and psychosocial summary score (PsS) were significantly lower than Dutch population norms (see table 4), except for the PsS at T1 during the dexamethasone free period. QoL was significantly more impaired during periods on dexamethasone for both PhS and PsS, and for most subscales. The clinical significance of these differences is reflected in mostly large effect sizes (see table 4). Over time, QoL became more impaired for some aspects as measured by the CHQ. At T2 during periods on dexamethasone, children scored worse on the physical summary scale (*d *= 0.57) and the subscales of family cohesion (*d *= 0.67) and emotional/behavioural role limitation (*d *= 0.58). Scores on the subscale of physical functioning decreased from T1 to T2, regardless of being on (*d *= 0.65) or off (*d *= 0.42) dexamethasone. Only a few differences between both risk groups were noted. Children in the High Risk protocol scored significantly lower on the CHQ the behaviour subscale at T2 during both periods (on dexamethasone p = 0.03 and off dexamethasone p = 0.04). Parental emotional impact was also stronger (i.e. lower CHQ score) at T2 for the High Risk group (both on and off dexamethasone p < 0.05, respectively).

**Table 4:** Child Health Questionnaire Parent Form 50

	T1 (n = 15)				T2 (n = 37)			
	Dexa + Mean (SD)	Dexa – Mean (SD)	Effect Size^1^	p^2^	Dexa + Mean (SD)	Dexa – Mean (SD)	Effect size^1^	p^3^
Physical Functioning	63.3# (15.0)	82.2# (13.3)	-1.26	<0.01	47.8#^(24.0)	72.4#^(23.6)	-1.03	<0.01
Role Limitations: emotional/behaviour	60.7# (33.6)	93.7 (9.5)	-0.98	0.01	41.1#^(30.2)	76.3# (25.1)	-1.17	<0.01
Role Limitations: physical	51.1# (20.4)	85.7# (14.4)	-1.70	<0.01	43.2# (25.0)	69.4# (29.5)	-0.89	<0.01
Bodily Pain	56.0# (18.4)	66.7# (16.3)	-0.58	0.05	43.8# (18.0)	64.3# (22.3)	-0.92	<0.01
General Behaviour	59.5# (16.6)	76.9 (16.9)	-1.03	0.01	55.8# (14.0)	68.9# (13.8)	-0.94	<0.01
Mental Health	59.7# (14.7)	74.7# (11.3)	-1.02	<0.01	55.3# (17.0)	68.9# (14.2)	-0.8	<0.01
Self-esteem	60.3# (15.2)	71.1# (13.4)	-0.71	0.01	54.4# (20.3)	66.3# (16.9)	-0.59	<0.01
General Health Perception	45.0# (19.1)	49.3# (12.2)	-0.23	NS	43.5# (20.8)	46.4# (21.0)	-0.14	NS
Parental Impact: emotional	54.4# (24.0)	62.8# (19.1)	-0.35	NS	50.7# (24.4)	54.3# (26.7)	-0.13	NS
Parental Impact: time	51.9# (27.1)	77.0# (17.1)	-0.93	0.01	43.8# (30.9)	60.4# (31.8)	-0.52	<0.01
Family Activities	56.3# (23.8)	75.8# (13.6)	-0.82	<0.01	41.7# (23.5)	58.6# (20.0)	-0.72	<0.01
Family Cohesion	74.7 (22.0)	75.0 (19.1)	-0.01	NS	59.2#^(23.2)	67.2 (17.4)	-0.34	<0.01
Physical Summary Score Z-score‡	30.6# (10.0)	42.2# (6.8)	-1.16	<0.01	24.3#^(11.1)	33.5# (13.2)	-0.70	<0.01
Psychosocial Summary Score Z-score‡	39.0# (11.4)	51.0 (5.1)	-1.05	0.01	34.5# (11.2)	44.4# (8.9)	-0.88	<0.01

Higher scores indicate a better QoL

^1 ^Effect size = negative effect size indicates worse QoL in the group on dexamethasone. Positive effect size indicates better QoL in the group on dexamethasone: 0.2 ≤ *d *< 0.5 = small effect; 0.5 ≤ *d *< 0.8 = moderate effect; *d *≥ 0.8 = large effect.

^2 ^Differences on and off dexamethasone, Wilcoxon signed ranks test

^3 ^Differences on and off dexamethasone, T-test for paired samples

# significant difference with Dutch reference (p < 0.05)

^Significant difference T1 vs. T2 (p < 0.05)

‡ Physical and Psychosocial CHQ summary scores based on a factor-analytical model on U.S. population samples. A score of 50 represents the mean in the general U.S. population. Scores below/above 50 are below/above the average in the general U.S. population.

## Discussion

This study demonstrated that children during treatment for ALL experience a reduced QoL, as compared to healthy children, which is further aggravated by the use of dexamethasone. This concords with results found in earlier studies on the effect of corticosteroids on children during cancer treatment. [13,15] Yet this is the first study to specifically assess the second year of ALL treatment and to include disease (i.e. cancer) specific QoL measures rather than only generic QoL questionnaires. This allows for more detailed information regarding the affected domains of QoL, including change over time and/or differences between relevant time points.

QoL was not only significantly lower than Dutch population norms, moreover children on active treatment for ALL also have a reduced QoL in comparison to children with other chronic diseases like asthma and, to a lesser extend, ADHD. [26,27] Similar results were found recently by Varni et al., in a comparative analysis of QoL in several disease clusters using the PedsQL 4.0 generic core scales. A large group of children with cancer (brain tumours, ALL, non-Hodgkin's lymphoma, Wilm's tumour, neuroblastoma and Hodgkin's lymphoma) and their parents reported significantly lower QoL in comparison to healthy children and a selection of other disease clusters. [28] In our study QoL was similar at 2 time points for most domains (see tables 2 and 4). For certain relevant domains however, QoL deteriorated over time. During maintenance treatment we found no improvement in QoL at T2 for any (sub)score, as was found by Eiser et al [15] during the first, most intensive, year of treatment. The decline in QoL for certain items during the second, less intensive year of treatment, might be related to an increasing cumulative dose of corticosteroids. This underscores the importance of continuing close monitoring and counselling of both child and family during the whole of treatment.

In HR children, reported scores on cognitive functioning were considerable lower than in NHR children, regardless of the use of dexamethasone. An important difference between both risk groups was total methotrexate dose and frequency of intrathecal therapy. Methotrexate has been associated with impaired neurocognitive functioning [29-31] and might explain the morbidity in cognition. Of course, no neuropsychological tests were performed and interpretation of these results should be done with care.

Limitations of this study are the small number of participants at T1. The introduction of a new protocol (ALL-10) at the start of this study limited the number of possible participants. Furthermore, the start of each dexamethasone period was accompanied by one dose of intravenous vincristine, followed by a second vincristine dose one week later. As vincristine is known to potentially have neurotoxic side-effects, it might interfere with certain aspects of QoL, such as pain and physical functioning. Hence an interaction between dexamethasone and vincristine on QoL can not be ruled out entirely. The study design (separate questionnaires referring to periods on and off dexamethasone) and parental counselling on potential side-effects of dexamethasone as part of usual care, might attend respondents to differences that would otherwise go unnoticed, causing bias. Although parents were instructed to assess both periods on and off dexamethasone independently, the response may have been flawed by sending the questionnaires in one single mailing and by the fact that parents were not strictly instructed to fill out questionnaires at the end of each period. In this study, parental and self rating of QoL did not differ statistically. The problem of proxy respondents is a widely debated issue in literature, with an overall consensus that children should be involved in QoL appraisal whenever possible. [32-34] If more self reports could have been obtained in this study, results might have been different. This might be addressed in future studies. However, since the peak incidence of childhood ALL is in young children, obtaining self reports will be a continuing problem in QoL studies during treatment.

Although it would have interesting to compare dexamethasone with prednisone, unfortunately the Dutch national ALL-9 treatment protocol does not allow for randomisation to different glucocorticosteroids and applied dexamethasone only.

## Conclusion

Although it has been suggested that impaired quality of life during cancer treatment in children is the effect of treatment as a whole, rather than the specific effect of various components (e.g. corticosteroids) [15], the results of this study suggest that dexamethasone probably plays an important (and possibly underestimated) role. The success of advancement in oncology is best illustrated with the reduction of mortality in childhood ALL. Therefore increasing attention can and should be given to the emotional burden of childhood ALL for both the patient and family. Treatment for ALL adversely affects all aspects of quality of life and, although the effect of other therapeutic agents can not entirely be ruled out, corticosteroids seem to have an additional negative effect. Counselling and coping of children and their parents with regard to the possible effects of corticosteroids is therefore essential to help them improve quality of life. The reduction of adverse effect of maintenance chemotherapy on QoL in childhood ALL in general, and of dexamethasone in particular, should therefore be subject of further studies without jeopardising the cure-rate.

## Competing interests

The authors declare that they have no competing interests.

## Authors' contributions

MV: coordination of the study, gathering and processing of data (questionnaires), performed the statistical analyses and drafting of manuscript. RL: coordination of the study, gathering and processing of data (questionnaires), performed the statistical analyses, drafting and revising of manuscript. JH: participated in the design of the study and helped to draft the manuscript. MG: participated in the coordination of the study, acquisition of data and helped to draft the manuscript. BV: participated in the coordination of the study, acquisition of data and helped to draft the manuscript.

GK: conceived the study, participated in its design and coordination, assisted the statistical analysis, helped to draft the manuscript. RG: conceived the study, participated in its design and coordination, assisted the statistical analysis, helped to draft and revise the manuscript. MV and RL have both contributed equally to the manuscript.

All authors read and approved the final manuscript.

## References

[B1] Eiser C, Vance YH, Horne B, Glaser A, Galvin H (2003). The value of the PedsQLTM in assessing quality of life in survivors of childhood cancer. Child Care Health Dev.

[B2] Eiser C, Eiser JR, Stride CB (2005). Quality of life in children newly diagnosed with cancer and their mothers. Health QualLife Outcomes.

[B3] Speechley KN, Barrera M, Shaw A, Morrison HI, Maunsell E (2006). Health related Quality of Life among child and adolescent survivors of childhood cancer. Journal of Clinical Oncology.

[B4] Waters EB, Wake MA, Hesketh KD, Ashley DM, Smibert E (2003). Health-related quality of life of children with acute lymphoblastic leukaemia: comparisons and correlations between parent and clinician reports. Int J Cancer.

[B5] Bostrom BC, Sensel MR, Sather HN, Gaynon PS, La MK, Johnston K, Erdmann GR, Gold S, Heerema NA, Hutchinson RJ, Provisor AJ, Trigg ME (2003). Dexamethasone versus prednisone and daily oral versus weekly intravenous mercaptopurine for patients with standard-risk acute lymphoblastic leukemia: A report from the Children's Cancer Group. Blood.

[B6] Gaynon PS, Trigg ME, Heerema NA, Sensel MG, Sather HN, Hammond GD, Bleyer WA (2000). Children's cancer group trials in childhood acute lymphoblastic leukemia: 1983–1995. Leukemia.

[B7] Veerman AJP, Hahlen K, Kamps WA, Van Leeuwen EF, De Vaan GAM, Solbu G, Suciu S, Van Wering ER, Berg AVDD Van den (1996). High cure rate with a moderately intensive treatment regimen in non-high-risk childhood acute lymphoblastic leukemia: Results of protocol ALL VI from the Dutch Childhood Leukemia Study Group. Journal of Clinical Oncology.

[B8] Jones B, Freeman AI, Shuster JJ, Jacquillat C, Weil M, Pochedly C, Sinks L, Chevalier L, Maurer HM, Koch K (1991). Lower incidence of meningeal leukemia when prednisone is replaced by dexamethasone in the treatment of acute lymphocytic leukemia. Medical and pediatric oncology.

[B9] Stuart FA, Segal TY, Keady S (2005). Adverse psychological effects of corticosteroids in children and adolescents. Archives of Disease in Childhood.

[B10] Soliday E, Grey S, Lande MB (1999). Behavioral effects of corticosteroids in steroid-sensitive nephrotic syndrome. Pediatrics.

[B11] Kayani SSDC (2002). Adverse behavioral effects of treatment for acute exacerbation of asthma in children. A comparison of two doses of oral steroids. Chest.

[B12] McGrath P, Pitcher L (2002). 'Enough is enough': qualitative findings on the impact of dexamethasone during reinduction/consolidation for paediatric acute lymphoblastic leukaemia. SupportCare Cancer.

[B13] Barr RD, Petrie C, Furlong W, Rothney M, Feeny D (1997). Health-related quality of life during post-induction chemotherapy in children with acute lymphoblastic leukemia in remission: An influence of corticosteroid therapy. International journal of oncology.

[B14] Hinds PS, Hockenberry MJ, Gattuso JS, Srivastava DK, Tong X, Jones H, West N, McCarthy KS, Sadeh A, Ash M, Fernandez C, Pui CH (2007). Dexamethasone alters sleep and fatigue in pediatric patients with acute lymphoblastic leukemia. Cancer.

[B15] Eiser C, Davies H, Jenney M, Stride C, Glaser A (2006). HRQOL implications of treatment with dexamethasone for children with acute lymphoblastic leukemia (ALL). PediatrBlood Cancer.

[B16] Raat H, Bonsel GJ, Essink-Bot ML, Landgraf JM, Gemke RJBJ (2002). Reliability and validity of comprehensive health status measures in children: The Child Health Questionnaire in relation to the Health Utilities Index. Journal of Clinical Epidemiology.

[B17] Landgraf JM, Abetz L, Ware JA (2006). The CHQ user's manual.

[B18] Barrera M, Gee C, Andrews GS, Armstrong CA, Saunders FE (2006). Health-related quality of life of children and adolescents prior to hematopoietic progenitor cell transplantation: Diagnosis and age effects. Pediatric Blood and Cancer.

[B19] Raat H, Landgraf J, Oostenbrink R, Moll H, Essink-Bot M (2007). Reliability and validity of the Infant and Toddler Quality of Life Questionnaire (ITQOL) in a general population and respiratory disease sample. Qual Life Res.

[B20] Felder-Puig R, di Gallo A, Waldenmair M, Norden P, Winter A, Gadner H, Topf R (2006). Health-related quality of life of pediatric patients receiving allogeneic stem cell or bone marrow transplantation: Results of a longitudinal, multi-center study. Bone Marrow Transplant.

[B21] Marchese VG, Chiarello LA, Lange BJ (2004). Effects of physical therapy intervention for children with acute lymphoblastic leukemia. Pediatr Blood Cancer.

[B22] Meeske K, Katz ER, Palmer SN, Burwinkle T, Varni JW (2004). Parent proxy-reported health-related quality of life and fatigue in pediatric patients diagnosed with brain tumors and acute lymphoblastic leukemia. Cancer.

[B23] Varni JW, Burwinkle TM, Katz ER, Meeske K, Dickinson P (2002). The PedsQL in pediatric cancer: reliability and validity of the Pediatric Quality of Life Inventory Generic Core Scales, Multidimensional Fatigue Scale, and Cancer Module. Cancer.

[B24] Cohen J (1977). Statistical power analysis for behavioral sciences.

[B25] Kazis LE, Anderson JJ, Meenan RF (1989). Effect sizes for interpreting changes in health status. Medical care.

[B26] Asmussen L, Olson LM, Grant EN, Landgraf JM, Fagan J, Weiss KB (2000). Use of the child health questionnaire in a sample of moderate and low-income inner-city children with asthma. Am J Respir Crit Care Med.

[B27] Rentz AM, Matza LS, Secnik K, Swensen A, Revicki DA (2005). Psychometric validation of the child health questionnaire (CHQ) in a sample of children and adolescents with attention-deficit/hyperactivity disorder. Quality of Life Research.

[B28] Varni JW, CA L, TM B (2007). Impaired health-related quality of life in children and adolescents with chronic conditions: a comparative analysis of 10 disease clusters and 33 disease categories/severities utilizing the PedsQL 4.0 Generic Core Scales. Health Qual Life Outcomes.

[B29] Ochs J, Mulhern R, Fairclough D, Parvey L, Whitaker J, Ch'ien L, Mauer A, Simone J (1991). Comparison of neuropsychologic functioning and clinical indicators of neurotoxicity in long-term survivors of childhood leukemia given cranial radiation or parenteral methotrexate: a prospective study. J Clin Oncol.

[B30] Giralt J, Ortega J, Olive T, Verges R, Forio I, Salvador L (1992). Long-term neuropsychologic sequelae of childhood leukemia: comparison of two CNS prophylactic regimens. Int J Radiat Oncol Biol Phys.

[B31] Kerr J, Berg S, Blaney S (2001). Intrathecal chemotherapy. Crit Rev Oncol Hematol.

[B32] Janse AJ, Gemke RJBJ, Uiterwaal CS, Tweel I Van der, Kimpen JLL, Sinnema G (2004). Quality of life; patients and doctors don't always agree: A meta analysis. Journal of Clinical Epidemiology.

[B33] Parsons SK, Barlow SE, Levy SL, Supran SE, Kaplan SH (1999). Health-related quality of life in pediatric bone marrow transplant survivors: according to whom?. Int J Cancer Suppl.

[B34] Theunissen NC, Vogels TG, Koopman HM, Verrips GH, Zwinderman KA, Verloove-Vanhorick SP, Wit JM (1998). The proxy problem: child report versus parent report in health-related quality of life research. Quality of Life Research.

